# Brazilian national bariatric registry - pilot study

**DOI:** 10.1590/0100-6991e-20233382-en

**Published:** 2023-02-17

**Authors:** LYZ BEZERRA SILVA, LUIZ GUSTAVO DE QUADROS, JOSEMBERG MARINS CAMPOS, MARCOS LEÃO VILLAS BOAS, JOÃO CAETANO MARCHESINI, ÁLVARO ANTONIO BANDEIRA FERRAZ, ROBERTO LUIZ KAISER, ALEXANDRE AMADO ELIAS, RICARDO VITOR, LUIZ CLAUDIO CHAVES, ALMINO CARDOSO RAMOS

**Affiliations:** 1 - Universidade Federal de Pernambuco, Departamento de Cirurgia - Recife - PE - Brasil; 2 - Hospital Santa Joana Recife, Centro de Obesidade e Diabetes - Recife - PE - Brasil; 3 - Faculdade de Medicina do ABC - São Caetano - SP - Brasil; 4 - Faculdade de Medicina de Ribeirão Preto - Ribeirão Preto - SP - Brasil; 5 - Hospital Santo Amaro - Salvador - BA - Brasil; 6 - Clínica Baros - Salvador - BA - Brasil; 7 - Clínica Marchesini - Curitiba - PR - Brasil; 8 - Kaiser Hospital - São José do Rio Preto - SP - Brasil; 9 - Instituto Garrido - São Paulo - SP - Brasil; 10 - Hospital Alemão Oswaldo Cruz - São Paulo - SP - Brasil; 11 - Universidade Federal do Pará - Belém - PA - Brasil; 12 - Hospital Ophir Loyola - Belém - PA - Brasil; 13 - GastroObesoCenter - Metabolic Optimization Institute - São Paulo - SP - Brasil

**Keywords:** Bariatric Surgery, Registries, Obesity, Quality Indicators, Health Care, Cirurgia Bariátrica, Obesidade, Registros, Base de Dados

## Abstract

**Introduction:**

Brazil is a world leader in bariatric surgery. However, the actual number of surgeries performed in the country is still unknown. It is necessary to implement an instrument to monitor the quality of care provided. This study evaluated the implementation of a Bariatric Surgery Data Registry in Brazil.

**Methodology:**

the registry was developed with Dendrite Clinical Systems Ltd., with data collected prospectively on an internet-based software. Seven centers were selected based on surgical volume and data entry commitment. The project covered three years after system implementation.

**Results:**

1,363 procedures performed by 17 surgeons were included. Most patients were female (67.2%), with average age of 39 years old and average baseline BMI of 41.5kg/m^2^. Diabetes mellitus was present in 34.5%, and hypertension in 40.1%. Roux-en-Y gastric bypass was performed in 79.3%, 95.5% by laparoscopy. There was one in-hospital death of cardiovascular cause. The average hospital stay was 2.03 days. The surgery-related complication rate was 0.97% in the first month, with three reoperations. Short-term follow-up was recorded in 75.6% and one-year follow-up in 21.64%. Total body weight loss was 10% in 30 days, rising to 33.3% after one year, with no difference between surgical techniques.

**Conclusions:**

the population profile was in accordance with the global registry of the International Federation for the Surgery of Obesity and Metabolic Disorders. The main difficulty encountered was low postoperative data entry. The experience acquired in this project will help advance data collection and knowledge of the safety and effectiveness of bariatric surgery in Brazil.

## RESUMO

**Introdução:**

o Brasil é líder mundial em cirurgia bariátrica. No entanto, o número de cirurgias realizadas no país ainda é desconhecido. É necessário um instrumento para monitorar a qualidade da assistência prestada. Este estudo avaliou a implantação de um Registro de Dados de Cirurgia Bariátrica no Brasil.

**Metodologia:**

O registro foi desenvolvido com a Dendrite Clinical Systems Ltd., com dados coletados prospectivamente em software na internet. Sete centros foram selecionados, baseado no volume cirúrgico e compromisso de inserção de dados. O projeto abrangeu três anos após implementação.

**Resultados:**

foram incluídos 1.363 procedimentos realizados por 17 cirurgiões. A maioria dos pacientes eram mulheres (67,2%), com idade média de 39 anos e IMC basal médio de 41,5kg/m^2^. Diabetes mellitus estava presente em 34,5% e hipertensão em 40,1%. Bypass gástrico em Y de Roux foi realizado em 79,3%, 95,5% por laparoscopia. Houve um óbito hospitalar de causa cardiovascular. A média de permanência hospitalar foi de 2,03 dias. A taxa de complicações relacionadas à cirurgia foi 0,97% no primeiro mês, com três reoperações. Seguimento de curto prazo foi registrado em 75,6% e de um ano em 21,64%. A perda de peso corporal total foi de 10% em 30 dias, aumentando para 33,3% após um ano, sem diferença entre técnicas cirúrgicas.

**Conclusões:**

o perfil populacional está de acordo com o registro global da IFSO. A principal dificuldade encontrada foi a baixa inserção de dados pós-operatórios. A experiência adquirida neste projeto ajudará no conhecimento da segurança e eficácia da cirurgia bariátrica no Brasil.

## INTRODUCTION

The growth of obesity rates, and the safety and efficacy of bariatric surgery, has contributed to the significant increase in procedures performed annually^1^ ^,^ ^2^. Access to bariatric surgery is variable worldwide, and despite the growth in numbers, it is still performed in only a small fraction of eligible patients^3^ ^,^ ^5^. Overall, there is little knowledge about the demographics and trends of patients undergoing bariatric surgery^2^ ^,^ ^6^ ^,^ ^7^. 

According to the International Federation of Surgery for Obesity and Metabolic Diseases (IFSO) registry report, presented in 2019 in Madrid, 833,687 surgical procedures were registered from 61 countries. These numbers, however, do not reflect the total number of operations worldwide since participating in this registry is optional^8^. The Brazilian Society of Bariatric and Metabolic Surgery (SBCBM) estimates that over 68,000 surgeries are performed annually in the country^9^. However, this number is not precisely known, as there is no national data record. 

National bariatric surgery registries have been successfully implemented in a few countries^10^ ^-^ ^13^. In Sweden, the bariatric registry covers the entire public and private health network, with data from over 70,000 procedures^14^. Reports are published once a year with 98% accuracy, covering around 99% of the procedures performed. This database is used to plan government strategies and for the publication of several important studies^10^ ^,^ ^15^ ^-^ ^19^. The same platform is used in other countries, such as United Kingdom, Australia, Germany, Turkey, and Kuwait.

Registries usually aim to assess the quality of care, and in the specific case of bariatric surgery, they can provide data on long-term surgical effects on weight and comorbidities^10^. Brazil, one of the countries with the largest volume of bariatric surgeries globally, has a fundamental role in disseminating knowledge. A national registry on bariatric surgery from SBCBM will monitor and reflect the status of bariatric surgery in the country. The data collected will provide real-world information on the number of surgeries, techniques employed, the profile of the population, and rates of success and complications.

## METHODS

The Brazilian National Pilot Registry was implemented in seven bariatric reference centers, selected by SBCBM based on surgical volume, expertise, and commitment to data input. The centers combined were expected to include a minimum of 1,000 patients per study year. Herein, we provide data considering short-term outcomes of three years after the system implementation. 

In partnership with Dendrite Ltd., models of bariatric surgery registries used in other countries were evaluated, and a protocol of variables to be collected was created, translated into Portuguese, and adapted to the Brazilian reality. After the development of the system, data entry began in 2017.

Data were inserted into the online database (Dendrite Intellect Web National Registry), confidentially maintained in the system, and stored in a private secured server. Data were collected prospectively during the hospital stay and routine follow-up visits. An active search was done to increase the follow-up rate. Post-operative data were collected after one month, six months, and one year. Each center designated a member to be trained for data input, and a national coordinator was available to solve any queries and check for inconsistencies. 

The primary objective was to acquire knowledge on the profile of bariatric surgeries performed in the selected centers. Specific data included demographics, surgical technique, complications, and duration of hospital stay. Post-operative follow-up included weight loss and comorbidities control. The secondary objective was to determine the feasibility of implementing a national registry in the country.

This research counted on financial support from the Investigator-Initiated Study Program of Ethicon Inc., a Johnson & Johnson Company, represented in Brazil by Johnson & Johnson do Brasil Indústria e Comércio de Produtos para Saúde Ltda. (Grant no. 15-607). All centers obtained local Ethics Committee approval. The statistical analysis was performed descriptively, based on data provided by the system in Excel tables. Two independent analyses were performed. The first was provided directly by Dendrite Inc. using an automatic data analysis tool. After that, manual statistics were performed to verify data.

## RESULTS

The registry included 1363 individual operative reports at the time of the last data submission. A total of seven centers, 17 surgeons, and 25 hospitals were included.

There were 1,320 procedures registered as primary operations (not revisions or planned 2^nd^ step), which are the focus of this analysis. Of these, short-term follow-up data (15-90 days) were registered for 1031 cases (75.6%). One-year follow-up was available for 295 cases (21.64%) (Table 1).

**Table 1:** Number of procedures registered.

Center	Performed surgeries	15-90 days	3-7 months	7-10 months	10-14 months	>14 months
Clínica Kaiser	306	298	190	92	141	64
Clínica Marchesini	91	48	18	0	0	0
Gastrobeso Center	290	190	137	56	43	12
Hosp. Alemão Oswaldo Cruz	246	178	26	5	2	0
Hospital Ophyr Loyola	68	22	4	0	0	1
Instituto Garrido	177	150	6	4	77	7
UFPE/COD-HSJR	185	145	65	45	32	55
Total	1363	1031	446	202	295	139

### Operation Type

**Figure 1:**
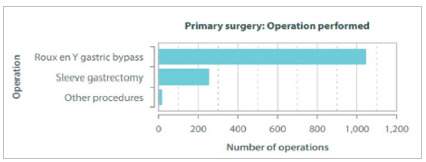
Types of procedures performed.

Most procedures were Roux-en-Y-Gastric Bypass (RYGB) (79.3%), performed laparoscopically (95.5%) (Figure 1). Most of the open procedures (n=60) were performed in public health system hospitals. Additional / concomitant procedures were done in 91 cases, mostly cholecystectomies (n=34) and hernioplasties (n=30). Private health insurances provided financing in 78.3%, self-pay in 11.1%, and public system coverage corresponded to 7.2%.

### Demographic Data

**Figure 2:**
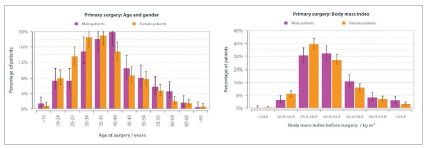
Distribution of cases by age / gender and BMI.

The overall proportion of female patients was 67.2% (n=915). Most patients were between 30 to 44 years old (n=688, 52.12%) at the time of the operation. The mean age was 39 years (SD: 11.2 years). The mean baseline body mass index (BMI) was 41.5 kg/m^2^ (SD: 6.9 kg/m^2^). Only 118 patients (8.9%) had a BMI greater than 50 kg/m^2^. In four cases, the BMI was under 30, and in 98, between 30.1-34.9 kg/m^2^ (Figure 2).

### Preoperative Comorbidities

**Figure 3:**
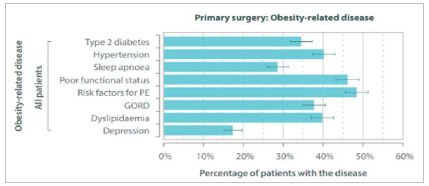
General prevalence of comorbidities.

As expected, a large proportion of patients had obesity-related diseases (Figure 3). Overall, those classified as having type 2 diabetes accounted for 34.5% of patients (predominantly male - 40.2%); 40.1% had hypertension (52.8% in males). Most of the patients with diabetes were treated only with oral antidiabetics, and 2.5% were under insulin.

### Short-Term Follow Up

For the 1,363 primary operations, 75.64% had short-term follow-up data entered in the database. Mean hospital stay was 2.03 days (0.5-9 days). 

The postoperative surgery-related complication rate in the first 30 days was 0.97%, mostly bleeding (n=8), and one case of a gastric leak at the His angle in an RYGB, treated by laparoscopy. The in-hospital mortality rate was 0.07% (n=1), secondary to an acute myocardial infarction. No surgery-related deaths were registered. The number of reoperations in the first 30 days was low (n=3, 0.24%) as expected for specialized high-volume centers. There were 2 reoperations in RYGB and 1 in sleeve gastrectomy (SG). The 30-day surgery-related complication rate was 0.45% for cardiovascular causes and 0.9% for other complications. (Table 2) Most common non-cardiac complications were common biliary duct stones/cholangitis (n=4), followed by vomiting/electrolyte disturbance (n=3). Other reported complications were pneumonia/atelectasis (n=2) and unanticipated transfer to ICU (n=2).

**Table 2:** General rates of complications.

		Complication recorded		
		No	Yes	%
30-day operative complications	RYGB	982	8	0.81%
	SG	239	4	1.65%
	All	1221	12	0.97%
Cardiovascular complication	RYGB	929	5	0.54%
	SG	165	0	0.00%
	All	1112	5	0.45%
Other complications	RYGB	923	7	0.75%
	SG	162	3	1.82%
	All	1102	10	0.90%

### Weight loss and Comorbidities

**Figure 4:**
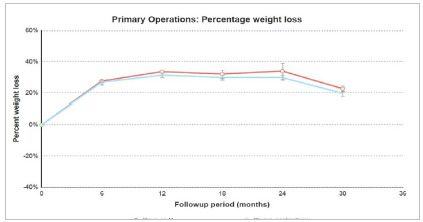
Postoperative weight loss by procedure.

Percentage weight loss at 30 days was 10.0% of total body weight-loss (TBWL) (SD 4.2%). The average TBWL was 27.3% for RYGB; 25.6% for sleeve gastrectomy in 6 months follow-up. After 1-year, TBWL was 33.5% for RYGB and 32.1% for SG (33.3% when considering both techniques) (Figure 4).

## DISCUSSION

According to SBCBM, 68,530 bariatric surgeries were performed in Brazil in 2019, a 7% increase compared to the previous year^11^. However, this represents only 0.5% of the eligible population^11^. The estimated number of procedures performed yearly is still imprecise, based on data from centers of excellence reported to SBCBM. There is no reliable report on the number of bariatric procedures performed annually in the private health system. The objectives of this study were to evaluate the feasibility of implementing a bariatric registry in Brazil and collect data on the profile of surgeries performed in selected centers. This knowledge is of great value for understanding the current scenario, preparing new strategies to improve access to surgical care, and serving as a source of information for patients, surgeons, and payors.

Creating a quality registry is justified when new technologies are introduced, and an existing intervention is increasingly used. Bariatric surgery fits well into those two criteria. National registries are an effective way of collecting data on bariatric and metabolic surgery. Large numbers can be reached if participating centers have a real commitment to include all operated patients, demonstrating the outcomes and benefits after the operations. The culture of regularly submitting data may improve bariatric and metabolic surgery acceptance by private and public payors^20^. 

Active participation in registries is usually voluntary and may reflect the experience of centers of excellence, and in many instances, not allowing generalization to the general population in many cases^10^. The main reason is that the population can vary between centers, especially in a country of continental dimensions like Brazil. In addition, high-volume centers usually have a lower rate of complications and better outcomes. This is a pilot registry involving seven centers. Therefore, the data analyzed consists of a sample and may not be representative of the country. However, we see consistency in the population profile and general outcomes compared to the most recent data from the global IFSO registry^8^. This reinforces the internal validity of the Brazilian pilot registry.

In this study, 67.2% of the population was female, with an average age of 39 years old and an average baseline BMI of 41.5 kg/m^2^. In IFSO’s data, 77.1% were female, with an average age of 43 years and average preoperative BMI of 44.3 kg/m^2^.^8^. In the Kuwait report, 73.6% of the population was female, with an average age of 32.6 years, and an average BMI of 45.9 kg/m^2^ for men and 43.4 kg/m^2^ for women^21^. 

Regarding comorbidities, in the IFSO report, 23.3% of patients were using medication for diabetes, and 41% for hypertension. In addition, 16.5% were being treated for depression, 18.9% had sleep apnea, and 25.1% had gastroesophageal reflux disease (GERD) 8. In the present study, 34.5% were classified as having diabetes, 40.1% with hypertension, 17.3% treated for depression, 28.6% with sleep apnea, and 37.7% with GERD. 

In the global registry, most of the procedures were SG, followed by RYGB. Brazil is one of the countries with the highest rates of RYGB (76.6%). More than 99% of the cases were performed by laparoscopy, figures that could not have been predicted 20 years ago when obesity was usually a contraindication for laparoscopic surgery^8^. In our registry, RYGB was the technique of choice in 79.3% of cases. In some countries, the profile of surgeries performed is different. In Kuwait, most procedures are SG, followed by single anastomosis gastric bypass (OAGB), and only about 1% are RYGB^21^. In Israel, OAGB went from 0.1% in 2014 to 46.1% in 2018, becoming the most performed surgery that year. On the other hand, SG fell from 80% in 2014 to 37% in 2018. RYGB remains constant as 10% of all operations performed^22^. 

The average length of stay in IFSO data was 2.1 days for RYGB and 1.9 days for SG^8^. In Sweden, Norway, the Netherlands, and Brazil, the average hospital stay is less than two days, while in some other countries, longer than five days^8^. In our study, the average hospital stay was 2 days. The data conform with what is shown in the world report. It is important to note that there is a greater adherence to fast-track hospital discharge protocols in centers of excellence.

The low morbidity and mortality shown in this study probably reflect that the selected centers are considered of excellence, with high surgical volume and highly skilled teams. The in-hospital mortality rate was 0.07% (n=1) due to an acute myocardial infarction. There were no deaths directly related to the surgery. There were only 3 reoperations during the first 30 days. The rate of postoperative complications surgery-related in the first 30 days was 0.97%. Complication or mortality rates were not assessed in the IFSO registry.

One of the main struggles in implementing a data registry in Brazil is the large number of surgeons and bariatric teams in the country. In addition, each surgeon may be involved in more than one team, usually performing surgery in several different hospitals due to health insurance policies. Data mining may be difficult because of the different medical record charts in hospital networks, whether traditional paper or electronic.

Even though participation by all accredited centers in the country is mandatory in the Israeli registry, they face the same problems regarding follow-up data. In the last report, the one-year follow-up was less than 50%, dropping to less than 20% after three years of surgery^22^. The follow-up in our registry may have been limited by the common practice of not inserting the data concurrently with the postoperative visit. Of the 1,363 cases, only 32.7% recorded data after six months and 21.64% in one year.

In the IFSO report, only 30.1% of cases had one or more follow-up records. The average one-year weight loss was 31.1%. After this period, 64.2% of patients using antidiabetic medication no longer needed them. There were similar reductions in the use of hypertension drugs and statins^8^. In this study, weight loss after one year was 33.3% TBWL. Few patients had their comorbidity data included in the one-year follow-up, which hindered a more accurate evolution analysis.

The most significant limitation of the present study is that it does not include all surgeries performed in the centers in the specified period. This can lead to a selection bias and does not provide data related to the total surgical volume of the centers and the country. In addition, data are entered by the surgeon himself or a designated member of the team, which can lead to bias and a lower rate of reported complications. Despite being in line with other registries, the low follow-up may also reflect difficulty collecting data by the centers due to the multiplicity of clinical records. In a future national implementation of a bariatric surgery registry, the difficulties faced in this pilot study should be considered. A more simplified platform design may be more suitable, with fewer collected variables, mainly reducing those related to specific surgical and postoperative technique data. In addition, integration with systems where long-term data is entered by the patient himself could be considered.

The registry implementation was successful in the 7 selected centers, considering the adequate operation of the platform and the successful data insertion by the centers. One of the main obstacles encountered was the follow-up data entry, which may reflect a low follow-up rate and a lower adherence to the registry after the initial surgical period. The population’s demographic profile is compatible with what is observed in worldwide registries, and surgical outcomes were as expected for the centers selected. Undoubtedly, the experience acquired in the Brazilian pilot registry project will help advance data collection and knowledge of the portrait of bariatric surgery in Brazil.
